# Headaches and Magnesium: Mechanisms, Bioavailability, Therapeutic Efficacy and Potential Advantage of Magnesium Pidolate

**DOI:** 10.3390/nu12092660

**Published:** 2020-08-31

**Authors:** Jeanette A. Maier, Gisele Pickering, Elena Giacomoni, Alessandra Cazzaniga, Paolo Pellegrino

**Affiliations:** 1 Dipartimento di Scienze Biomediche e Cliniche L. Sacco, Università di Milano, 20157 Milano, Italy; alessandra.cazzaniga@unimi.it; 2 Department of Clinical Pharmacology, University Hospital and Inserm 1107 Fundamental and Clinical Pharmacology of Pain, Medical Faculty, F-63000 Clermont-Ferrand, France; gisele.pickering@uca.fr; 3 Sanofi Consumer Health Care, 20158 Milan, Italy; Elena.Giacomoni@sanofi.com (E.G.); Paolo.Pellegrino@sanofi.com (P.P.)

**Keywords:** magnesium, pidolate, deficiency, headache, migraine, BBB

## Abstract

Magnesium deficiency may occur for several reasons, such as inadequate intake or increased gastrointestinal or renal loss. A large body of literature suggests a relationship between magnesium deficiency and mild and moderate tension-type headaches and migraines. A number of double-blind randomized placebo-controlled trials have shown that magnesium is efficacious in relieving headaches and have led to the recommendation of oral magnesium for headache relief in several national and international guidelines. Among several magnesium salts available to treat magnesium deficiency, magnesium pidolate may have high bioavailability and good penetration at the intracellular level. Here, we discuss the cellular and molecular effects of magnesium deficiency in the brain and the clinical evidence supporting the use of magnesium for the treatment of headaches and migraines.

## 1. Background

A large body of literature suggests a relationship between magnesium deficiency and mild and moderate tension-type headaches and migraines [1,2,3,4,5,6,7,8,9]. The International Classification of Headache Disorders (ICHD-3-beta) divides all headache entities into primary and secondary disorders [10] and approximately 90% of headaches seen in general practice are of the primary variety, such as migraine, tension-type headache, or cluster headache [11]. Magnesium for headaches offers an alternative to traditional medication that brings with it issues, such as addiction and side effects. Magnesium, with its relative lack of side effects, is particularly compelling for use in groups in which side effects are less well tolerated, such as children, pregnant women and the elderly population.

Magnesium is the fourth most abundant cation in the human body [12,13] and is involved in several important functions, such as enzyme activity, oxidative phosphorylation, DNA and protein synthesis, neuromuscular excitability and parathyroid hormone secretion [14].

**Figure 1:**
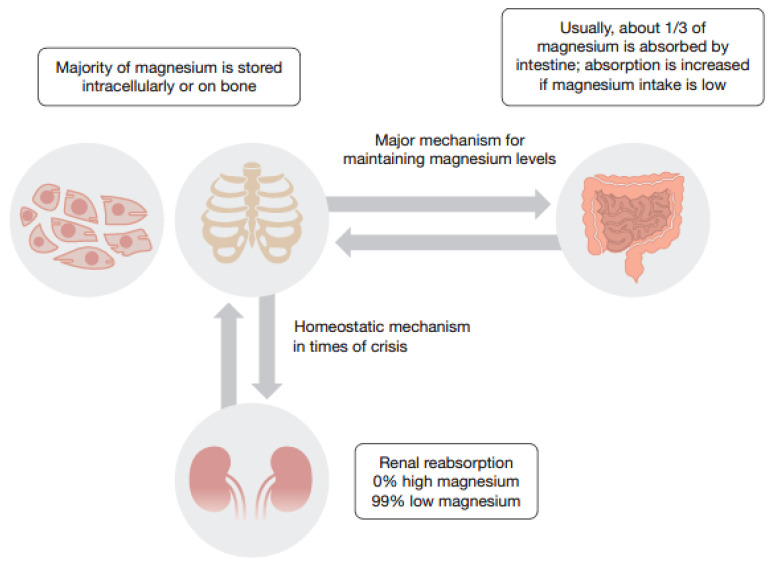
Schematic presentation of magnesium homeostasis.

Approximately 99% of total body magnesium is stored intracellularly in soft tissue and muscle (~40%) or resides as a component of bone on the surface of hydroxyapatite crystals (~60%) [15,16,17]. The absorption of magnesium occurs predominantly in the small intestine (and to a lesser extent in the colon) and depends on two different pathways: a passive paracellular transport, which facilitates bulk magnesium absorption, and an active transcellular pathway responsible for mediating the fine-tuning of magnesium absorption [18]. In the kidney, 80% of total serum magnesium is filtered in the glomeruli, with more than 95% being re-absorbed in the nephron. The renal re-absorption of magnesium contributes to maintaining magnesium homeostasis, as it declines to near zero in the presence of high levels of magnesium and reaches over 99% in the presence of magnesium depletion (Figure 1) [19]. Serum magnesium concentration is strictly regulated by the balance between intestinal absorption, renal excretion and bone buffer (Figure 1).

Magnesium is surrounded by two hydration shells. Consequently, the radius of hydrated magnesium is about 400 times larger than its dehydrated radius. This creates steric constraints for magnesium transporters, which need to dehydrate magnesium, an event that is highly energy consuming, before transferring it through the membrane [12]. Over the last 20 years, several putative magnesium channels and transporters have been described [20], but the workings of intracellular magnesium homeostasis remain a conundrum.

Magnesium deficiency may occur for several reasons: inadequate intake, gastrointestinal loss and renal loss, or re-distribution from the extracellular to the intracellular space. Acute magnesium deficiency may be asymptomatic or associated with various disorders, such as nausea, vomiting, lethargy, [19] nervousness/anxiety and stress [21,22]. Chronic deficiency may lead to severe neuromuscular and cardiovascular pathologies [19]. There are multiple studies that suggest a relationship between magnesium deficiency and headaches, and these will be discussed further in the text [1,2,3,4,5,6,7,8,9]. Challenges exist for measuring magnesium concentration [23], and standardized laboratory tests that accurately evaluate magnesium levels are lacking [24]. Currently, in adults the reference interval for serum magnesium ranges between 0.75–0.95 mmol/L (1.82–2.30 mg/dL), while serum ionized magnesium ranges between 0.50–0.69 mmol/L [19,25]. These values are based on data reported in the 1970s [26]. However, since serum magnesium respond to dietary manipulation [23], and magnesium content in fruits, cereals and vegetables markedly declined over the past 40 years [24], the distribution of serum magnesium in normal population should be updated. In addition, serum magnesium concentration is most often used to assess magnesium status yet only 1% of total body magnesium is present in blood. In some instances, magnesium deficiency may be masked as the large proportion of magnesium residing in bone provides a large exchangeable pool to buffer changes in serum magnesium concentration [16]. For example, in an analysis carried out in women with normal serum values, a significantly greater magnesium retention was shown in osteoporotic patients compared with healthy individuals, thus suggesting the presence of magnesium deficiency despite normal magnesium serum values [27]. The magnesium load test, which analyzes urine samples over 24 h, is currently used to measure whole body magnesium, although it can prove difficult to administer as measurements are taken over 24 h in order to take account of circadian rhythms [28]. The ionized magnesium of erythrocyte cells can also be used as a measure of total body magnesium as, among intracellular magnesium compartments, erythrocytes make up more than 90% of the total blood cells, therefore mainly affect the intracellular blood magnesium content [29].

Magnesium salts used in current clinical practice to treat magnesium deficiency can be organic, such as magnesium pidolate and magnesium lactate, or inorganic, such as magnesium chloride and magnesium carbonate (Table 1). Different salts have been noted to have varying absorption efficiency and soluble properties, leading to a variation in bioavailability.

Magnesium pidolate may have high bioavailability [35,36] and good penetration at the intracellular level [37]. Furthermore, magnesium pidolate is able to reverse magnesium deficiency responsible for headaches, even after a short administration period [31], and to prevent pediatric tension-type headaches [38]. Taking this into consideration, the unique mechanism of action of magnesium pidolate and the efficacy and safety of magnesium salts for the treatment of headaches is considered.

## 2. Why Should Magnesium Be Used to Treat Headaches?

Multiple studies have suggested a relationship between magnesium deficiency and headaches (Table 2) [8]. In a case-control study of patients suffering from migraine, reduced magnesium levels were found in serum [7], cerebrospinal fluid [1] and the ictal and interictal regions within the brain [2]. Similar results were observed in several other case-control studies [4,5,6,8,9]. For example, Sarchielli and colleagues have shown that migraine sufferers with and without aura and tension-type headaches have significantly lower levels of serum and salivary magnesium [8]. Importantly, a study by Trauninger and colleagues using the magnesium load test revealed a greater retention of magnesium in patients suffering from migraines compared with healthy controls, suggesting a systemic magnesium deficiency associated with migraine [6]. Furthermore, a 2-week trial revealed that, when 29 migraine patients took mineral water containing 110 mg/L magnesium daily, their total magnesium in erythrocytes significantly increased, compared with 18 healthy controls [4]. A recent observation by Assarzadegan and colleagues [9] indicated that a decrease in magnesium levels in serum increased the odds of acute migraine headaches by a factor of 35 in 40 patients with migraine versus 40 healthy controls, and that magnesium deficiency is an independent risk factor in the incidence of migraines. Studies carried out by Mauskop and colleagues [3,39] estimated the frequency of magnesium deficiency among migraine sufferers by evaluating the efficacy of the intravenous infusion of 1 g of magnesium sulfate for the treatment of patients with headaches. They investigated the correlation of clinical responses and basal serum ionized magnesium level and reported that a 50% reduction in pain was noted after infusion [3,39]. Taken together, these results suggest a correlation between magnesium deficiency and headaches, and of note, they suggest that magnesium deficiency represents an independent risk factor for migraine occurrence.

The intravenous infusion of magnesium sulfate as a treatment for acute headaches was assessed in a systematic review with varying results [40]. Initial efficacy was demonstrated in adults with low serum magnesium [3,39], and a small study confirmed that the treatment was safe in adolescents [41]. A recent systematic review indicated no benefit immediately after infusion, but potential benefits in pain control beyond the first hour [40]. There is, however, a counterargument that only a proportion of patients with acute headaches have magnesium deficiency [39], which may mask the extent of the therapeutic effectiveness of magnesium infusion in the emergency setting [42].

Magnesium can act as a calcium channel antagonist in neurons, where it is believed to prevent the excessive activation of the excitatory synapses (e.g., *N*-methyl-d-aspartate [NMDA] receptors); it has also been shown to downregulate inflammation through inhibiting pro-inflammatory intracellular signaling, such as the nuclear factor kappa B pathway [43]. Of interest, magnesium homeostasis in the brain has been found to be dysregulated in various neurological disorders [44]. Lower concentrations of magnesium than in healthy controls were found in the brains of patients with Alzheimer’s and Parkinson’s diseases [44] and in the occipital lobes of patients with migraine and cluster headaches [45]. In magnesium-deficient individuals, magnesium supplementation attenuates anxiety and stress symptoms [23,46]. Similarly, magnesium-deficient mice exhibit an anxiety-related behavior, which is due, in part, to the increased response of the hypothalamic–pituitary–adrenal axis, the central stress response system [47].

**Figure 2:**
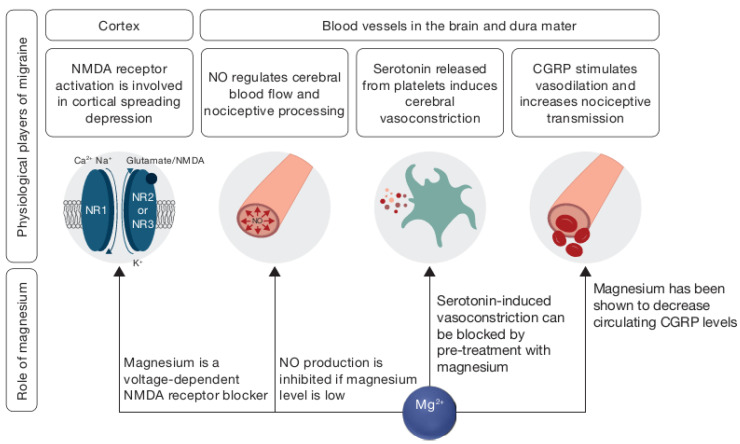
Mechanisms involved in migraine and possible role of magnesium. CGRP, circulating calcitonin gene-related peptide; NMDA, N-methyl-D-aspartate; NO, nitric oxide.

A number of mechanisms have been described to explain the relationship between magnesium deficiency and headaches (Figure 2) [48]. Magnesium deficiency has been associated with cortical spreading depression (CSD), thought to be responsible for the aura associated with migraines [48], imbalanced neurotransmitter release [49], platelet activity [50] and vasoconstriction [51]. In CSD, substance P, a neuropeptide which acts as a neurotransmitter and neuromodulator, is released as a result of magnesium deficiency, possibly acting on sensory fibers and producing headache pain [52]. Magnesium has also been shown to decrease the level of circulating calcitonin gene-related peptide (CGRP), which is involved in migraine pathogenesis through its ability to dilate intracranial blood vessels and produce nociceptive stimuli [48,53]. External magnesium may help to diminish various aspects of neurogenic inflammation as it is involved in the control of NMDA glutamate receptors, which play an important role in pain transmission within the nervous system [54], the regulation of cerebral blood flow [55] and the initiation and spread of CSD. It has been shown that ionized magnesium can block CSD by regulating glutamatergic neurotransmission, closing the NMDA receptor calcium channel and modulating the cyclic adenosine monophosphate (cAMP) response element-binding protein signaling [56,57]. The modulation of the cerebral blood flow by circulating nitric oxide (NO) is one of the mechanisms involved in headaches, and it has been shown to be influenced by magnesium intake [48,58]. Magnesium can also increase vasodilation directly through blocking calcium-sensitive potassium channels on smooth muscle cells [59]. There is some evidence that magnesium may be most beneficial in migraines with aura [60,61].

Another key molecule in migraine pathogenesis is serotonin, a potent cerebral vasoconstrictor released from platelets during a migraine attack—it also promotes nausea and vomiting [62]. A decrease in serum ionized magnesium level and an elevation of the serum ratio of ionized calcium to ionized magnesium may increase the likelihood for cerebral vascular muscle serotonin receptor sites, potentiate cerebral vasoconstriction induced by serotonin and facilitate serotonin release from neuronal storage sites [62]. Vasoconstriction induced by serotonin can be blocked by pretreatment with ionized magnesium [63].

## 3. Magnesium Supplementation—Therapeutic Efficacy

The therapeutic efficacy of magnesium supplementation in headache patients has been shown in two double-blind, placebo-controlled randomized trials [31,32]. The first study was conducted in 20 women with menstrual migraine. It is known that the magnesium level of erythrocytes and leukocytes of women with premenstrual syndrome is lower than that in the women without the syndrome [64]. For this reason, magnesium supplementation is widely used to treat premenstrual syndrome [31,65,66]. Women received two cycles of 360 mg of magnesium pyrrolidone carboxylic acid or placebo taken daily from ovulation to the first day of their period. Patients receiving active treatment had a significant reduction in the frequency of headaches and total pain index [31]. A larger double-blind, placebo-controlled randomized study of 81 adult patients with migraines, according to the International Headache Society (IHS) criteria, also showed significant improvements in patients on active therapy [32]. The active group received 600 mg of trimagnesium dicitrate in a water-soluble granular powder every morning and had a significant reduction (*p* < 0.05) in the frequency of attacks (41.6%) compared with the placebo group (15.8%). A further randomized controlled trial of 118 children 3–17 years of age receiving 9 mg/kg daily oral magnesium oxide or placebo showed that treatment led to a significant reduction in headache days [67].

One trial, enrolling 69 patients taking 242 mg magnesium-u-aspartate-hydrochloride-trihydrate daily, showed no effect on migraines [33]. Diarrhea occurred in almost half of the 35 patients receiving magnesium compared with a quarter of the 34 patients on placebo indicating that the magnesium salt may be poorly absorbed, which may account for the observed lack of efficacy.

The duration of 1500 mg daily oral magnesium pidolate treatment needed to normalize serum magnesium levels was investigated by Aloisi and colleagues in a study on 40 children designed to evaluate the correlation between magnesium deficiency and the effect on visual evoked potentials. The analysis showed that a treatment lasting 20 days was sufficient to normalize serum magnesium levels in 90% of treated patients [68].

Koseoglu and colleagues evaluated the prophylactic effects of 600 mg daily oral magnesium citrate supplementation in 30 migraine patients without aura compared with 10 patients on placebo treatment. Migraine attack frequency, severity, and P1 amplitude (in visual evoked potential examination) decreased after magnesium treatment compared with pretreatment values and placebo [69].

Karimi and colleagues, in a randomized, double-blind, controlled, crossover trial, gave 63 patients oral daily 500 mg magnesium oxide followed by 800 mg valproate sodium (400 mg every 12 h) or vice versa for 24 weeks. Patients showed a similar number and mean duration of migraine attacks in both groups, indicating that magnesium oxide is as effective as valproate in migraine prophylaxis without significant adverse effects [70].

A recent systematic review of five randomized, double-blind, placebo-controlled trials in adult migraine patients showed possible evidence for the prevention of migraines with 600 mg magnesium dicitrate daily, and that it is a well-tolerated and cost efficient strategy in clinical use [71].

In view of the results of these studies, several national and international guidelines added the recommendation of oral magnesium for headache patients [72,73,74]. The Italian Headache Society (SISC) guideline mentions magnesium pidolate supplementation for menstrual migraine and pre-menstrual syndrome patients, but a precise administration schedule has not been established [74]. Notably, magnesium pidolate is used at much higher concentrations than other magnesium salts (Table 3).

Side effects were not measured in all studies, but in those that were, diarrhoea and gastric effects were the most common, although mild in all instances, and did not prevent patients from completing treatment [32,33,69,70,75]. See Table 3 for studies describing the efficacy and safety of magnesium in treating headache symptoms.

## 4. Magnesium Salt Biovailability—Pidolate Versus Other Salts

Magnesium pidolate is an organic salt and, based on animal studies, may have a high bioavailability [35,36]. The bioavailability of magnesium is of high importance in treating headaches as the more magnesium that can be absorbed, the more effective the treatment. In a study by Coudray and colleagues in rats, absorption was 13% higher from organic than inorganic magnesium salts and particularly high urinary excretion with magnesium gluconate and pidolate was observed [35]. Magnesium pidolate exhibited higher bioavailability compared with other organic salts in mice: the post-oral serum magnesium increase was higher in mice receiving magnesium pidolate (100% versus baseline) than in mice treated with magnesium lactate (50% versus baseline) [36]. Other magnesium salts have been studied in a limited number of studies in humans conducted in the early 1990s, with mixed results. In some studies, there was no difference between organic and inorganic magnesium salts [76,77,78,79]; others demonstrated slightly higher bioavailability of organic magnesium salts under standardized conditions [18,34,80,81,82,83]. Magnesium pidolate is an organic salt and organic salts have been found to be consistently more bioavailable that inorganic salts in many human studies. Despite the lack of studies specifically analyzing magnesium pidolate bioavailability, it could be postulated that magnesium pidolate availability is, in part, due to its organic properties [18,34,80,81,82,83].

Magnesium pidolate has good intracellular penetration, which has been shown in vivo. Ten patients with sickle cell disease were treated with daily oral magnesium pidolate (540 mg/70 kg), which resulted in a reduced number of dense erythrocytes and improved erythrocyte membrane transport abnormalities in patients [37]. However, a recent review of the literature conducted by Zhang and colleagues failed to demonstrate any efficacy of the most common oral salts of magnesium [84]. The reason for this discrepancy may be due to differences in the ability of various salts to enter different cell lines. A recent study showed that the bioavailability at the cellular level of magnesium pidolate is different from that of two inorganic salts (magnesium chlorate and sulfate) in cell cultures of osteogenic sarcoma, which could suggest a lower capacity of magnesium pidolate to enter bone cells, the body’s main deposit for magnesium. This would explain the greater availability for other tissues and cells, such as lymphocytes and polymorphonuclear cells [85].

## 5. Magnesium Pidolate and Brain Penetration

The dysfunction of the blood–brain barrier (BBB) has been described in several neurological disorders, including ischemic stroke and inherited and neurodegenerative diseases [86,87]. This topic remains controversial: while some studies did not find changes in BBB during a migraine attack [88], there are studies in human subjects and animals that indicate that BBB permeability may be increased with migraine and headaches [89,90]. BBB disruption has been associated with magnesium deficiency in the brain [91,92]. It is therefore interesting to distinguish agents that exert a protective role on BBB and prevent its impairment in response to various challenges. There is evidence that magnesium has a protective role on the BBB in vivo [93,94], and a recent paper has highlighted that 10 mmol/L magnesium sulfate reduces the permeability in an in vitro model of the human BBB [95]. This effect could be the result of the antagonism between calcium and magnesium in the endothelial actin cytoskeleton, which remodels intercellular gap formation, thus inhibiting the paracellular movement of molecules through the tight junctions [96].

Romeo et al. (2019) [95] compared the effect of different magnesium salts at the same concentration (5 mmol/L) in in vitro in models of rat and human BBBs. All salts decreased BBB permeability; among them, magnesium pidolate and magnesium threonate were the most efficient in the rat model, and magnesium pidolate was the most efficient in the human model, suggesting differences in response between humans and rodents.

Another aspect evaluated in Romeo’s study [95] was that the transport of magnesium through the BBB is more efficient after magnesium pidolate treatment. Magnesium has been found to cross the intact BBB and enter the central nervous system in rats, to an extent proportional to magnesium serum levels [93,94]. In humans with an intact BBB, a modest but significant increase in magnesium concentration in the cerebrospinal fluid was reported after systemic administration of magnesium sulfate [97]. The use of magnesium pidolate may result in more magnesium crossing the BBB compared with other salts and, therefore, may have special relevance for the treatment of neurological conditions with a known connection to magnesium deficiency [95].

## 6. Magnesium Pidolate and Headache: A Challenge for the Future

Headache is characterized by high lifetime prevalence [98] and, rather than taking preventative medications [98], most patients use non-steroidal anti-inflammatory drugs (NSAIDs), mainly purchased in an over the counter setting without medical advice or prescription.

A literature evidence base suggests that magnesium deficiency increases the risk of headache. As shown in several clinical studies and reported in various national and international guidelines [72,73,74], the treatment of magnesium deficiency can reduce the frequency of headaches and, as a direct consequence, the use of NSAIDs and other therapies [93,94].

Magnesium pidolate has high bioavailability and good intracellular penetration [82] and it may reverse the magnesium deficiency responsible for headaches, even after a short administration period. Tissue culture and animal model studies indicate that magnesium pidolate may be slightly more effective than other magnesium salts in crossing the BBB [95], and magnesium in general is believed to exert neuroprotective functions. Further studies on the tissue distribution of magnesium pidolate may help to better understand its specific properties.

## 7. Conclusions

Taken together, these results confirm a correlation between magnesium deficiency and headaches. In addition, they suggest magnesium deficiency could be an independent risk factor for migraine occurrence. Some of the trials presented in this review date from the 1990s; however, it is encouraging to see a revitalization of this subject with more recent systematic reviews and clinical trials.

Magnesium deficiency is more often present in postmenopausal women with osteoporosis (84%) [99] and in women aged 18 to 22 (20%) [100]. The use of magnesium with its relatively low side effects is particularly pertinent for these populations who are also particularly susceptible to the side effects of traditional drugs.

When assessing the efficacy of magnesium salt, variations in dosage, study design, methods of assessment and study population, all need to be evaluated, which can make it difficult to interpret which salt is preferable for treating headache. In terms of magnesium pidolate, it may have a lower capacity to enter bone cells, the body’s main deposit for magnesium [82], and may cause more magnesium to cross the BBB compared with other salts [95]. Due to its potential high bioavailability, it may have special relevance for the treatment of neurological conditions with a known connection to magnesium deficiency, such as headache. Based on the information in the literature, there is an argument for the use of magnesium pidolate in Italy. However, it needs to be borne in mind that only a limited number of studies have shown the benefits of magnesium pidolate in headaches, and further controlled studies are needed. This is particularly important with regard to elucidating any side effects, as the 1500–4500 mg dose is high compared to the other salts, which range between 242 mg and 600 mg.

Overall, the use of oral magnesium salt represents a well-tolerated and inexpensive addition for the treatment of headache patients, to reduce the frequency of attacks and the costs of treatment both in terms of economic burden and adverse events.

## Figures and Tables

**Table 1:** Inorganic and organic salts used for magnesium supplementation [30,31,32,33,34].

Inorganic Magnesium Salts	Organic Magnesium Salts	Combinations/Different Formulations
Carbonate	Acetate	Citrate + hydrogen-l-glutamate
Chloride	Aspartate	Dicitrate
Oxide	Citrate	Glycinate lysinate chelate
Sulfate	Gluconate	Oxide + glycerophosphate
	Lactate	Pyrrolidone carboxylic acid
	Pidolate	Trimagnesium dicitrate
		U-aspartate-hydrochloride-trihydrate

**Table 2:** Studies investigating the relationship between magnesium levels and headache.

Year	Type of Headache	Number of Patients	Outcome	Reference
1985	Migraine	57 adults	Reduced magnesium levels in cerebrospinal fluid	[1]
1989	Migraine	11 adults	Reduced magnesium levels in the brain	[2]
1995	Cluster	22 adults	Up to 50% of migraine patients were found to be magnesium-deficient	[3]
2000	Migraine	29 adults plus 18 healthy controls	Total magnesium in erythrocytes significantly increased compared with healthy controls	[4]
2002	Tension/migraine	25 adults plus 20 healthy controls	Reduced magnesium levels in serum and saliva	[5]
2002	Migraine	20 adults plus 20 healthy controls	Increased systemic retention of magnesium vs. controls	[6]
2011	Migraine	140 adults plus 140 healthy controls	Total serum magnesium levels significantly lower vs. controls	[7]
2012	Migraine	50 adults plus 50 healthy controls	Total serum magnesium levels significantly lower vs. controls	[8]
2016	Acute migraine	40 adults plus 40 healthy controls	Decreased magnesium indicates a 35-fold increased risk of acute migraine	[9]

**Table 3:** Efficacy and safety of magnesium in treating headache symptoms.

Type of Study	Author/Year	Study Length	Country	Type of Headache	Number of Patients	Magnesium Salt	Efficacy Outcome	Safety Outcome	Reference
**Children**									
Multi-arm	Aloisi, 1997	20 days	Italy	Tension, migraine	60 male and female children 6–13 years	1500 mg daily oral magnesium pidolate	20 days treatment sufficiently normalizes serum Magnesium levels in 90% of migraine patients	NR	[68]
Double-blind, placebo-controlled randomized trial	Wang, 2003	16 weeks	USA	Migraine	118 male and female children 3–17 years (n = 60, placebo)	9 mg/kg daily oral magnesium oxide	Significant reduction in headache days	NR	[67]
Open label trial	Grazzi, 2007	3 months	Italy	Tension	45 male and female children 8–16 years	2250 mg x2 daily oral magnesium pidolate	Headache days decreased by 69.9%	No significant side effects	[38]
**Adults**									
Double-blind, controlled, randomized, crossover trial	Karimi, 2019	24 weeks	Iran	Migraine	63 adult male and females	500 mg daily oral magnesium oxide (800 mg sodium valproate)	Magnesium oxide appears to be as effective as valproate in migraine prophylaxis without significant adverse effects	No side effects on top of headache symptoms	[70]
Systematic review (five clinical trials below)	Von Luckner, 2018	2–4 months	Various countries	Migraine	Five clinical trials of adult male and females	Different salts different doses	Possibly effective in preventing migraine. Safe and cost efficient	NA	[71]
1. Double-blind, placebo-controlled randomized trial	Facchinetti, 1991	2 months	Italy	Menstrual migraine	20 females	360 mg daily oral magnesium pyrrolidone carboxylic acid	Significant reduction in the frequency of headache and total pain index	NR	[31]
2. Double-blind, placebo-controlled randomized trial	Peikert, 1996	12 weeks	Germany	Migraine	81 male and female adults (n = 38, placebo)	600 mg daily oral trimagnesium dicitrate	Significant improvement in patients on active therapy	Diarrhoea and gastric complaints (mild and tolerable)	[32]
3. Double-blind, placebo-controlled randomized trial	Pfaffenrath, 1996	12 weeks	Germany	Migraine	69 male and female adults (n = 34, placebo)	242 mg daily oral magnesium-u-aspartate-hydrochloride-trihydrate	No effect	Soft stool, diarrhoea (mild)	[33]
4. Double-blind, placebo-controlled randomized trial	Koseoglu, 2008	3 months	Turkey	Migraine	40 male and female adults (n = 10, placebo)	600 mg daily oral magnesium citrate	Migraine attack frequency, severity, and P1 amplitude decreased	Diarrhoea, soft stools, gastric irritation (mild)	[69]
5. Multicenter, crossover trial	Taubert, 1994	2 × 2 months	Germany	Migraine	63 adult male and females	600 mg daily oral trimagnesium dicitrate or placebo	Statistically significant reduction in the frequency of attacks compared with placebo	Diarrhoea	[75]

NA, not applicable; NR, not reported.
